# Particulate matter forecast and prediction in Curitiba using machine learning

**DOI:** 10.3389/fdata.2024.1412837

**Published:** 2024-05-30

**Authors:** Marianna Gonçalves Dias Chaves, Adriel Bilharva da Silva, Emílio Graciliano Ferreira Mercuri, Steffen Manfred Noe

**Affiliations:** ^1^Graduate Program of Environmental Engineering, Federal University of Paraná, Curitiba, Brazil; ^2^Perkons S.A., Curitiba, Brazil; ^3^Department of Environmental Engineering, Federal University of Paraná, Curitiba, Brazil; ^4^Institute of Forestry and Engineering, Estonian University of Life Sciences, Tartu, Estonia

**Keywords:** particulate matter, air pollution, vehicle emissions, optical sensor, neural network, Random Forest

## Abstract

**Introduction:**

Air quality is directly affected by pollutant emission from vehicles, especially in large cities and metropolitan areas or when there is no compliance check for vehicle emission standards. Particulate Matter (PM) is one of the pollutants emitted from fuel burning in internal combustion engines and remains suspended in the atmosphere, causing respiratory and cardiovascular health problems to the population. In this study, we analyzed the interaction between vehicular emissions, meteorological variables, and particulate matter concentrations in the lower atmosphere, presenting methods for predicting and forecasting PM2.5.

**Methods:**

Meteorological and vehicle flow data from the city of Curitiba, Brazil, and particulate matter concentration data from optical sensors installed in the city between 2020 and 2022 were organized in hourly and daily averages. Prediction and forecasting were based on two machine learning models: Random Forest (RF) and Long Short-Term Memory (LSTM) neural network. The baseline model for prediction was chosen as the Multiple Linear Regression (MLR) model, and for forecast, we used the naive estimation as baseline.

**Results:**

RF showed that on hourly and daily prediction scales, the planetary boundary layer height was the most important variable, followed by wind gust and wind velocity in hourly or daily cases, respectively. The highest PM prediction accuracy (99.37%) was found using the RF model on a daily scale. For forecasting, the highest accuracy was 99.71% using the LSTM model for 1-h forecast horizon with 5 h of previous data used as input variables.

**Discussion:**

The RF and LSTM models were able to improve prediction and forecasting compared with MLR and Naive, respectively. The LSTM was trained with data corresponding to the period of the COVID-19 pandemic (2020 and 2021) and was able to forecast the concentration of PM2.5 in 2022, in which the data show that there was greater circulation of vehicles and higher peaks in the concentration of PM2.5. Our results can help the physical understanding of factors influencing pollutant dispersion from vehicle emissions at the lower atmosphere in urban environment. This study supports the formulation of new government policies to mitigate the impact of vehicle emissions in large cities.

## 1 Introduction

Vehicle emissions represent one of the primary sources of air pollution in urban areas globally, with road traffic emissions constituting a significant portion of the particulate matter (PM) present, especially at the roadside (Charron et al., 2007). Particulate matter (PM) emissions from vehicles, which account for 56% of PM, encompass various sources, including exhaust emissions (Khazini et al., 2023). These emissions predominantly contribute to fine PM, known as PM_2.5_, which refers to particles with an aerodynamic diameter < 2.5 μm. Additionally, PM emissions arise from the re-suspension of dust and wear and tear of vehicle components such as brakes, tires, and clutches, primarily contributing to the coarse mode of PM (PM_2.5_ - PM_10_) (Abu-Allaban et al., 2003; Thorpe and Harrison, 2008; Kam et al., 2012; Pant and Harrison, 2013).

The population living in cities is exposed to high concentrations of PM, and the United Nations estimates that the world population living in urban areas will increase approximately 12% between 2022 and 2050 (United Nations, 2019). With this increase, the rise in vehicle fleets and the characteristics of cities will potentially affect the concentration of pollutants. The existence of a large number of buildings and scarcity of vegetation, associated with geographical and meteorological factors in urban environments, influence the diffusion, transformation, deposition, and removal of pollutants in the atmosphere (Abhijith et al., 2017; Harrison, 2018; Barwise and Kumar, 2020; Shakya et al., 2023).

Despite being known for its advances in urban mobility, Curitiba (Brazil) has observed an increase in the number of cars per inhabitant, which is higher than the population growth, and a decrease in the number of public transport users (Fochesatto et al., 2023). In 2023, the vehicle fleet in Curitiba was the fifth largest in the country, comprising more than 1.7 million vehicles, mostly cars, which accounted for approximately 66% of the fleet, followed by motorcycles, accounting approximately 10% of the fleet (BRASIL, 2023). Andrade et al. (2012) showed that the main sources of PM in Curitiba were vehicle emissions, which is responsible for most of the PM_2.5_ emitted. Mercuri et al. (2023) showed that the flow of vehicles in Curitiba is directly related to the concentration of particulate matter on urban roads.

Several studies have proposed models to predict PM concentration (Brokamp et al., 2018; Shang et al., 2019; Xiao et al., 2020), but identifying the key factors influencing these predictions remains a challenging problem. Prediction and forecasting are often used interchangeably, but in our research, the terms have a clear distinction. In this study, forecasting refers to the process of estimating fine particle concentration in the future based on past observation data. On the other hand, prediction refers to estimating PM_2.5_ concentration in the same time step as the input variables used to make the prediction.

The Random Forest algorithm is one of the most common models used for PM estimation. It has been increasingly used in studies predicting the concentration of atmospheric pollutants, with different temporal and spatial resolutions (Reichstein et al., 2019; Stafoggia et al., 2019; Xu et al., 2019), and it makes it possible to select variables of interest that can influence the concentration of PM_2.5_, calculating the importance of each one in the model and classifying them. Recently, there have been few studies using Recurrent Neural Networks (RNN) and their variations for air quality forecasting. Among these, long short-term memory (LSTM) takes into account the temporal dependencies in PM_2.5_ concentration records and has been increasingly applied (Bekkar et al., 2021; Dhakal et al., 2021; Guo et al., 2022).

Perez et al. (2020) used a neural network (NN) model and a linear model to predict the maximum 24-h PM_2.5_ average in Chile, and the authors found a higher accuracy using the neural network model. Hooyberghs et al. (2005) described the design of an NN prediction tool for ambient PM concentrations in Belgium; based on measurements from 10 monitoring sites from 1997 to 2001 and on simulations of meteorological parameters, they identified the boundary layer height (BLH) as the most important input variable. Li et al. (2020) evaluated and compared the performance of six common machine learning algorithms (MLAs), including Random Forest (RF), for predicting hourly street-level of PM_2.5_ concentrations at three roadside stations in Hong Kong, showing that RF was the MLA with the highest predictive accuracy and R^2^ values greater than or equal to 0.95. Kamińska (2018) applied RF to predict NO, NO_2_, and PM_2.5_ values in Wrocław, Poland. In the research, traffic volume, temporal characteristics, and meteorological conditions (wind speed and direction, temperature, pressure, and relative humidity) were considered as predictors; the author showed that in warmer periods, RF produces a better fit and that the most important predictors for PM_2.5_ concentrations were meteorological conditions, especially temperature and wind.

This study aims to use machine learning models (Random Forest and LSTM neural network) to estimate the concentration of PM_2.5_ in Curitiba, Brazil. The models to access PM_2.5_ concentrations were developed using data from optical sensors installed in the city between 2020 and 2022, meteorological variables, boundary layer height, vehicle flow count, and particulate matter concentration as input variables. We seek to identify the importance of different input variables and compare PM_2.5_ prediction and forecasting model performances. The study is organized as follows: Section 2 describes the data related to vehicle counting, PM_2.5_ concentration, meteorological conditions, and boundary layer height, presents an overview of the machine learning models and a description of performance evaluation metrics; Section 3 contains the modeling results and discussion; and Section 4 summarizes the conclusions.

## 2 Materials and methods

This section is divided in four parts: subsection 2.1) a description of the solution developed by the authors to measure PM_2.5_ concentrations and the dataset construction based on vehicle count, meteorological, and boundary layer height data; subsection 2.2) a description of the Random Forest model used for PM prediction; subsection 2.3) an overview of the LSTM Neural Network architecture applied for PM forecast; and subsection 2.4) the performance metrics applied to evaluate the quality of predictions.

### 2.1 Vehicle, meteorological, and particle data

**Figure 1:**
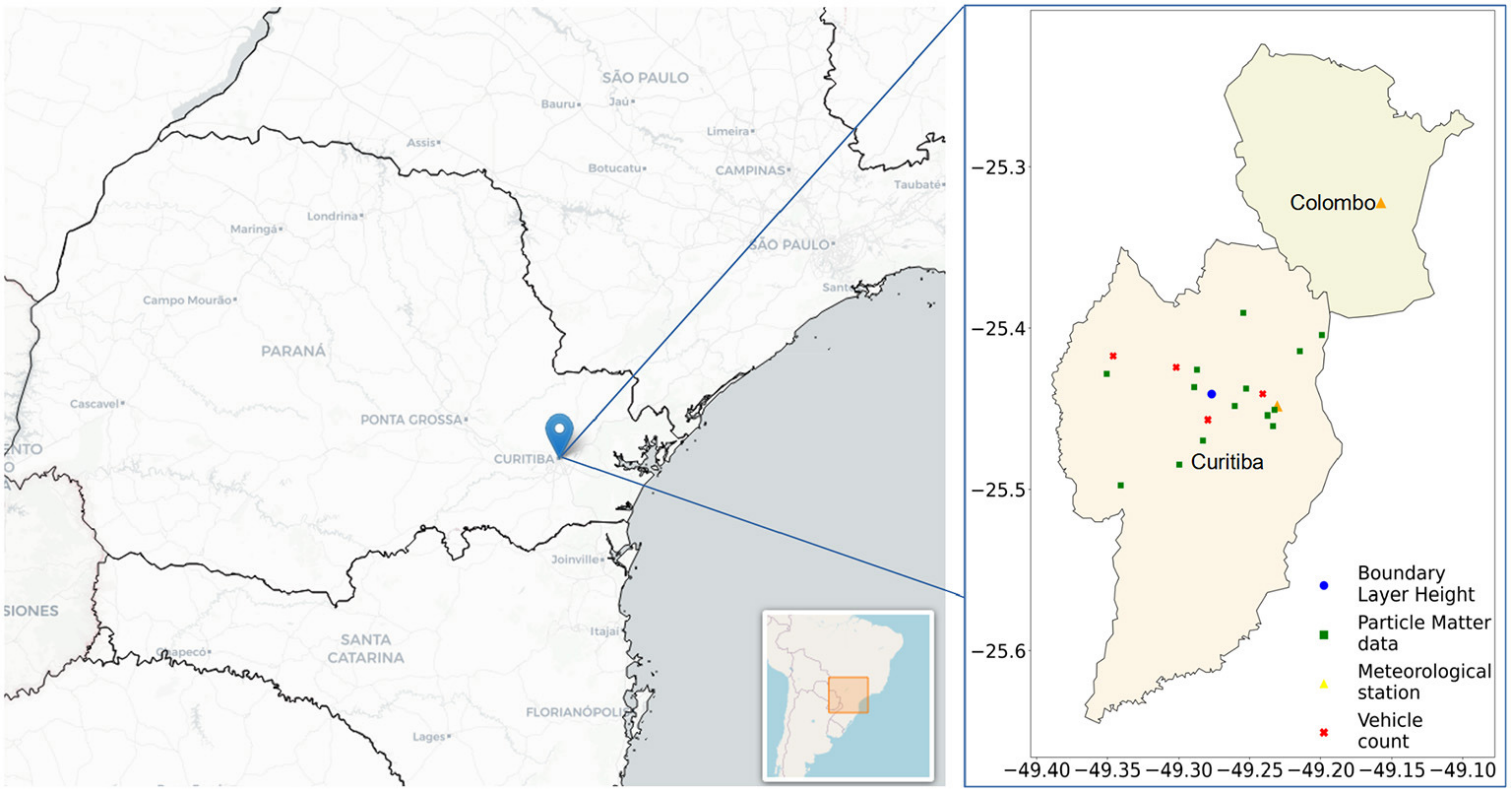
Location of SDS011 sensor points, vehicle count, meteorological stations, and ERA5 data in the city of Curitiba and metropolitan region (Colombo).

Particulate matter measurement was performed using an SDS011 sensor coupled to a Raspberry Pi single-board computer. This sensor employs optical technology and uses laser scattering to obtain the concentration of particulate matter between 0.3μm and 10μm, including inhalable particles classified as PM_2.5_ (World air quality index project, 2008). It represents a low-cost, low-power consumption measurement method with adaptability to different locations and climatic conditions, indicating the potential use for monitoring networks in various locations within a city or country (Liu et al., 2019; Tagle et al., 2020). The SDS011 sensors were deployed at 14 locations in the city of Curitiba (see Figure 1), which was characterized by predominantly paved streets and residential areas (Rodrigues et al., 2023, 2024). The data used in this study refer to the period from 1 January 2020 to 31 December 2022.

The vehicle count data were obtained from the Perkons company, covering four points in the city (Figure 1) from 1 January 2020 to 4 October 2022. The total vehicle count (including motorcycles, cars, and trucks) was used to represent the average hourly or daily vehicle count in the city. In addition to the number of vehicles, meteorological variables and the variation in the height of the boundary layer are expected to influence the concentration and dispersion of PM in the atmosphere. Therefore, air temperature (°C), relative humidity (%), atmospheric pressure (mB), global radiation (kJ m^−2^), wind speed (m/s), wind direction (°), wind gust (m/s), and precipitation data (mm) were obtained from the National Meteorological Institute (INMET) from two automatic weather stations: station A807 located at the Polytechnic Centre of the Federal University and station B806 located in the city of Colombo.

European Centre for Medium-Range Weather Forecasts (ECMWF) global climate atmospheric reanalysis data (ERA5) were utilized to obtain the variation in the planetary boundary layer height in Curitiba. The reanalysis combines model data with observations across the world, including satellite and radiosonde datasets and various observational datasets from the World Meteorological Organization's Global Telecommunication System (GTS) (Hersbach et al., 2020; Li et al., 2023). The ERA5 data cover the entire globe, on a 1440 × 721 grid with 0.25° latitude and 0.25° longitude resolution, a vertical resolution of 37 standard pressure layers, an hourly temporal resolution, and is computed by the bulk Richardson number method (a measure of the atmospheric conditions) (Hersbach et al., 2020; Guo et al., 2021). Figure 1 illustrates the location of Curitiba in Brazil and indicates the SDS011 sensor points, vehicle count locations, meteorological stations, and the site used for downloading ERA5 data.

### 2.2 PM_2.5_ prediction using Multiple Linear Regression and Random Forest

The Multiple Linear Regression (MLR) model was used as the baseline for predicting PM_2.5_. An MLR extends simple linear regression to include more than one explanatory variable, producing a multivariate model. The equation for the line in MLR modeling takes the form of Equation 1, where for *i* = *n* observations, *y*_*i*_ is the dependent variable, *x*_*i*_ is the explanatory variable, β_0_ is the y-intercept (constant term), β_*p*_ is the slope coefficients for each explanatory variable, and ϵ is the model's error term (also known as the residuals).


(1) $$\begin{array}{l} y_{i}=\beta_{0}+\beta_{1}x_{i1}+\beta_{2}x_{i2}+\ldots +\beta_{p}x_{ip}+\epsilon \end{array}$$

The Random Forest (RF) algorithm has been used to predict environmental variables concentrations, it is a classification and regression algorithm that integrates multiple decision trees through ensemble learning (Jeung et al., 2019). The RF model performs a random sampling of the original dataset using the decision tree as the basic random forest classifier resulting in *n* different sample datasets. These datasets are used to build *n* different decision tree models, with the final findings depending on the average value of these decision tree models (Kamińska, 2019; Luo et al., 2023). Essentially, the RF is constructed by a large number of trees, and the algorithm calculates the average result of all trees, as shown in Equation 2, where $\hat{f}\left(x\right)$ is the result of the RF non-linear regression, *K* is the number of trees, and *T*(*x*) is the result of each regression tree.


(2) $$\begin{array}{l} \hat{f}\left(x\right)=\frac{1}{K}\overset{K}{\underset{k=1}{\sum }}T\left(x\right) \end{array}$$

In this study, the RF model was created for predicting the hourly and daily mass concentrations of PM_2.5_ (dependent variable) using meteorological, vehicle count, and boundary layer height variables described in Section 2.1 as predictive (independent) variables. After calculating hourly and daily averages and cleaning missing data, the dataset was divided into 80% of training and 20% of test datasets. This division was made using a method to split arrays or matrices into random train and test subsets with a random state that controls the shuffling applied to the data before applying the split to ensure reproducible output across multiple function calls. A total of 1,000 decision trees were used to apply the random forest regression method, which is a meta estimator that fits a number of decision tree regressors on various sub-samples of the dataset and uses averaging to improve the predictive accuracy and control over-fitting. The performances of the MLR baseline estimation and RF model were calculated and compared for hourly and daily averages, as well as the importance of each predictor variable in the RF model.

### 2.3 PM_2.5_ forecast using naive model and long short-term memory neural network

The long short-term memory (LSTM) is one of the Recurrent Neural Network (RNN) models most widely used in air quality forecasting because it considers the temporal dependencies observed in PM_2.5_ concentration time series (Huang and Kuo, 2018; Bekkar et al., 2021). It was created to solve problems of long-term dependencies, which general RNNs cannot learn, and gradient vanishing or explosion in backpropagation, which means that the learning speed of the previous hidden layers is slower than the deeper hidden layers in RNNs, even leading to a decrease in accuracy rate as hidden layers increase (Huang and Kuo, 2018; Yadav et al., 2020; Bekkar et al., 2021). Meanwhile, LSTM has longer memory and can learn from inputs that are separated from each other by long time lags (Bekkar et al., 2021).

An LSTM has three analogical gates based on the sigmoid function, which works on the range between 0 and 1. The input gate controls the writing of input information, the forget gate determines whether the information is saved or released from the memory at each decision point, and the output gate decides what information to output (Huang and Kuo, 2018; Bekkar et al., 2021). To compare LSTM network's performance, Naive prediction's performance was build and used as reference. Naive forecasting models are based on the repetition of a historical observation solely, without trying to explain the underlying causal relationships that produce the variable being estimated (Shim et al., 2011; Ciechulski and Osowski, 2024). Our version of Naive model considers the forecast equal to the latest observation in a time series (Gleser, 1990), which means that the PM_2.5_ concentration was taken as the same as the previous hour (or day) on the current hour (or day).

Following the same procedure as for the RF model, missing data were cleaned, hourly and daily averages were calculated, and 20% of data were used in Naive's forecasting representing the test data, comprising the period from 17 March 2022 to 4 October 2022. The forecast errors were calculated and used as the reference for LSTM model, as described in the next section of performance metrics. The target for the LSTM model was PM_2.5_ concentration values at the subsequent timestep, i.e., the forecast horizon was set to 1 h or 1 day. We have varied the number of timesteps for the LSTM to look backward (window size) while predicting from 1 to 35. We have tested and compared different window sizes and hidden units while predicting, and the first 80% of the time series was used for training and the last 20% of data was used for testing the model. The LSTM final architecture has 2 and 3 hidden layers with 64 neurons each for hourly and daily models, respectively.

Data were preprocessed using a method to standardize features by removing the mean and scaling to unit variance. A standard LSTM code was written and optimized using the *PyTorch* package; we have used mean squared error (squared L2 norm) for the loss function and Stochastic Gradient Descent for the optimizer. The LSTM performance errors were calculated and compared with Naive's model.

### 2.4 Performance evaluation metrics

The mean absolute error (MAE), mean absolute percentage error (MAPE), root mean square error (RMSE), coefficient of determination (*R*^2^), and an accuracy metric were used to assess the prediction and forecast quality and compare the results of MLR with RF and of Naive estimation with LSTM. In Equations 3–6 below, *n* is the sample size, *o*_*i*_ and *p*_*i*_ represent the measured and predicted value, respectively, and ō denotes the mean of all measured values.

MAE (mean absolute error) is the arithmetic mean of the absolute deviations between the measured and predicted values of the sample, as shown in Equation 3.


(3) $$\begin{array}{l} \text{MAE}=\frac{1}{n}\overset{n}{\underset{i=1}{\sum }}|p_{i}-o_{i}| \end{array}$$

The mean absolute percentage error (MAPE) expresses the prediction or forecast error as a percentage and can be calculated from Equation 4.


(4) $$\begin{array}{l} \text{MAPE}=\frac{1}{n}\left(\overset{n}{\underset{i=1}{\sum }}|\frac{p_{i}-o_{i}}{o_{i}}|\right)100 \end{array}$$

RMSE (root mean square error) of a sample is the quadratic mean of the differences between the observed values and predicted ones. It reflects the prediction accuracy and its calculation formula is shown in Equation 5.


(5) $$\begin{array}{l} \text{RMSE}=\sqrt{\frac{1}{n}\overset{n}{\underset{i=1}{\sum }}{\left(p_{i}-o_{i}\right)}^{2}} \end{array}$$

The coefficient of determination (*R*^2^) reflects the proportion of all variations of the dependent variable that can be explained by the independent variable through the regression relationship and can be calculated by Equation 6.


(6) $$\begin{array}{l} R^{2}=1-\frac{\overset{n}{\underset{i=1}{\sum }}{\left(o_{i}-p_{i}\right)}^{2}}{\overset{n}{\underset{i=1}{\sum }}{\left(o_{i}-ō\right)}^{2}} \end{array}$$

The accuracy metric (ACC) is a percentage value, which depends on the MAPE and is calculated using Equation 7.


(7) $$\begin{array}{l} \text{ACC}=100-\text{MAPE} \end{array}$$

## 3 Results and discussion

**Figure 2:**
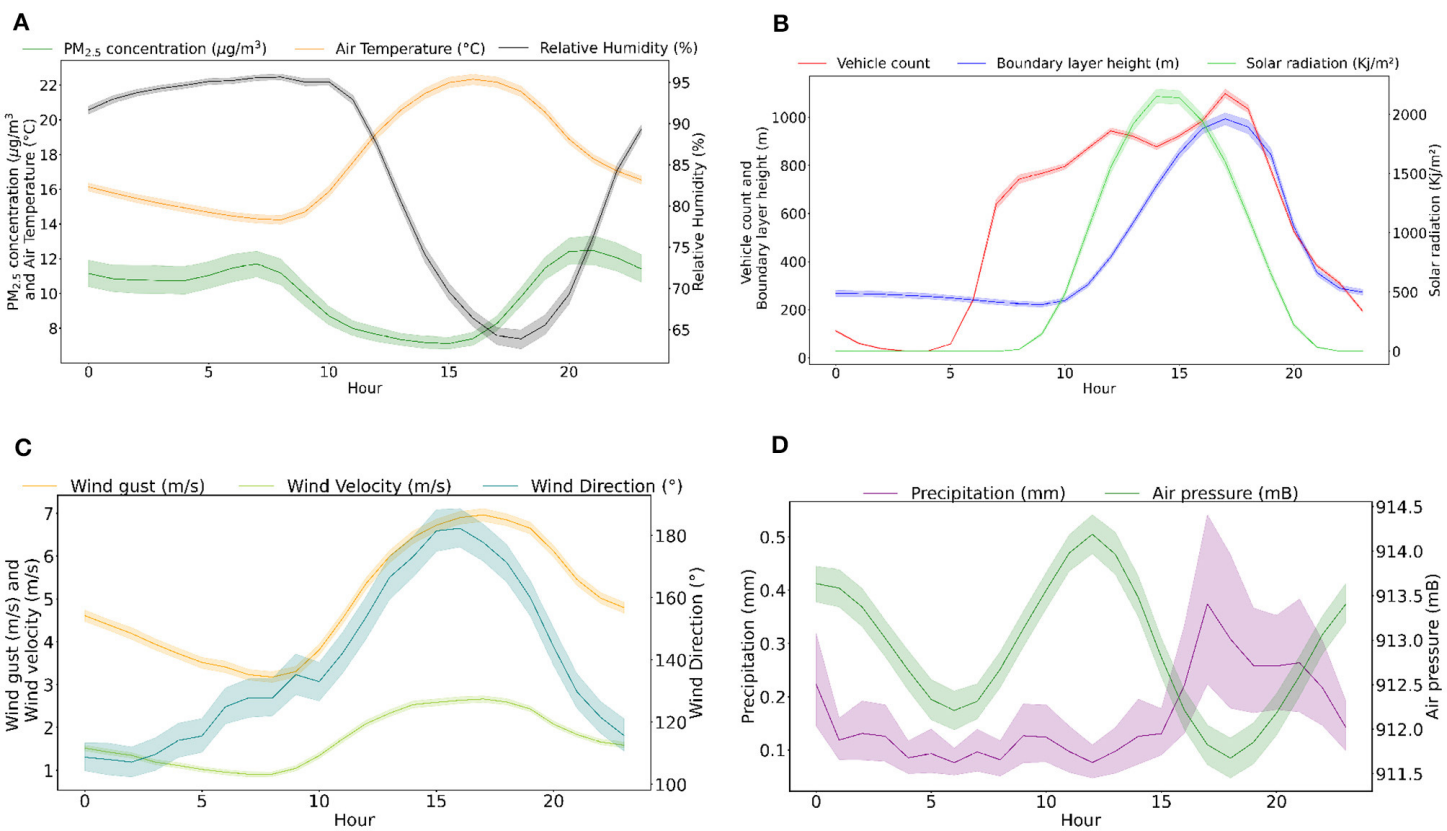
Daily profile with hourly averages of the dataset variables: **(A)** PM_2.5_ concentration, air temperature, and relative humidity; **(B)** vehicle count, BLH, and solar radiation; **(C)** wind gust, wind velocity, and wind direction; **(D)** precipitation and air pressure. Shaded areas represent confidence interval of 95%.

Figure 2 shows the daily profile of each variable with the lines representing the average in each hour of the day. From Figure 2B, it can be observed that the number of vehicles is an important source of particle emission in Curitiba, and that the PM_2.5_ concentration has one peak approximately 7 a.m. and another near 8 p.m. We note the effect of solar radiation in heating the surface, generating more dispersion and, consequently, vertical air mass movement, as represented by the increased wind velocity during the day. Changes in relative humidity and winds may also affect particle dynamics.

Multiple linear regression (MLR) for hourly time scale PM_2.5_ prediction, which is indicated by the dependent variable PM${\;}_{2.5}^{H}$ (μg/m^3^), is shown in Equation 8. The following independent variables from Equation 8 are hourly averages: *T*^*H*^ is air temperature (°C), *U*^*H*^ is relative humidity (%), $W_{s}^{H}$ is wind speed (m/s), $W_{d}^{H}$ is wind direction (°), $W_{g}^{H}$ wind gust (m/s), *R*^*H*^ is global radiation (kJ m^−2^), $P_{a}^{H}$ is atmospheric pressure (mB), *P*^*H*^ is precipitation data (mm), *V*^*H*^ is vehicle count, and *H*^*H*^ is planetary boundary layer height (m).


(8) $$\begin{array}{r} {\text{PM}}_{2.5}^{H}=+62.2933-0.3691T^{H}-0.2896U^{H}\\ -0.3037W_{s}^{H}+0.0080W_{d}^{H}-1.1688W_{g}^{H}-0.0011R^{H}\\ -0.0147P_{a}^{H}+0.0868P^{H}+0.0020V^{H}-0.0070H^{H} \end{array}$$

MLR for daily time scale prediction of PM_2.5_ concentration, which is indicated by the dependent variable PM${\;}_{2.5}^{D}$ (μg/m^3^), is described by Equation 9. The following independent variables from Equation 9 are daily averages: *T*^*D*^ is air temperature (°C), *U*^*H*^ is relative humidity (%), $W_{s}^{D}$ is wind speed (m/s), $W_{d}^{D}$ is wind direction (°), $W_{g}^{D}$ wind gust (m/s), *R*^*D*^ is global radiation (kJ m^−2^), $P_{a}^{D}$ is atmospheric pressure (mB), *P*^*D*^ is precipitation data (mm), *V*^*D*^ is vehicle count, and *H*^*D*^ is planetary boundary layer height (m).


(9) $$\begin{array}{r} {\text{PM}}_{2.5}^{D}=-25.5351-0.3300T^{D}\\ -0.4206U^{D}-4.0462W_{s}^{D}+0.0162W_{d}^{D}\\ +0.5726W_{g}^{D}-0.0041R^{D}+0.0881P_{a}^{D}-0.0635P^{D}\\ +0.0006V^{D}-0.0194H^{D} \end{array}$$

Table 1 summarizes the errors for test data using hourly and daily averages for the studied models (Random Forest and Multiple Linear Regression used for prediction, LSTM, and Naive models used for forecasting). All forecasts shown in Table 1 used window size equal to one (hour or day) and PM_2.5_ concentration as input data.

**Table 1:** Summary of prediction and forecast performance results.

**Model**	**MAE (μg/m^3^)**	**MAPE (%)**	**RMSE (μg/m^3^)**	**R^2^ (-)**	**ACC (%)**
**Prediction**					
MLR (hourly)	6.65	1.39	9.93	0.22	98.61
RF (hourly)	5.06	0.93	7.79	0.52	99.07
MLR (daily)	4.99	0.70	6.89	0.46	99.30
RF (daily)	4.33	0.63	6.21	0.56	99.37
**Forecast**					
Naive (hourly)	3.46	0.29	5.46	0.88	99.70
LSTM (hourly)	3.60	0.31	6.02	0.86	99.69
Naive (daily)	7.22	0.56	9.65	0.41	99.44
LSTM (daily)	6.82	0.52	9.03	0.39	99.48

Forecasts used window size equal to one (hour or day) and PM_2.5_ concentration as input data.

**Figure 3:**
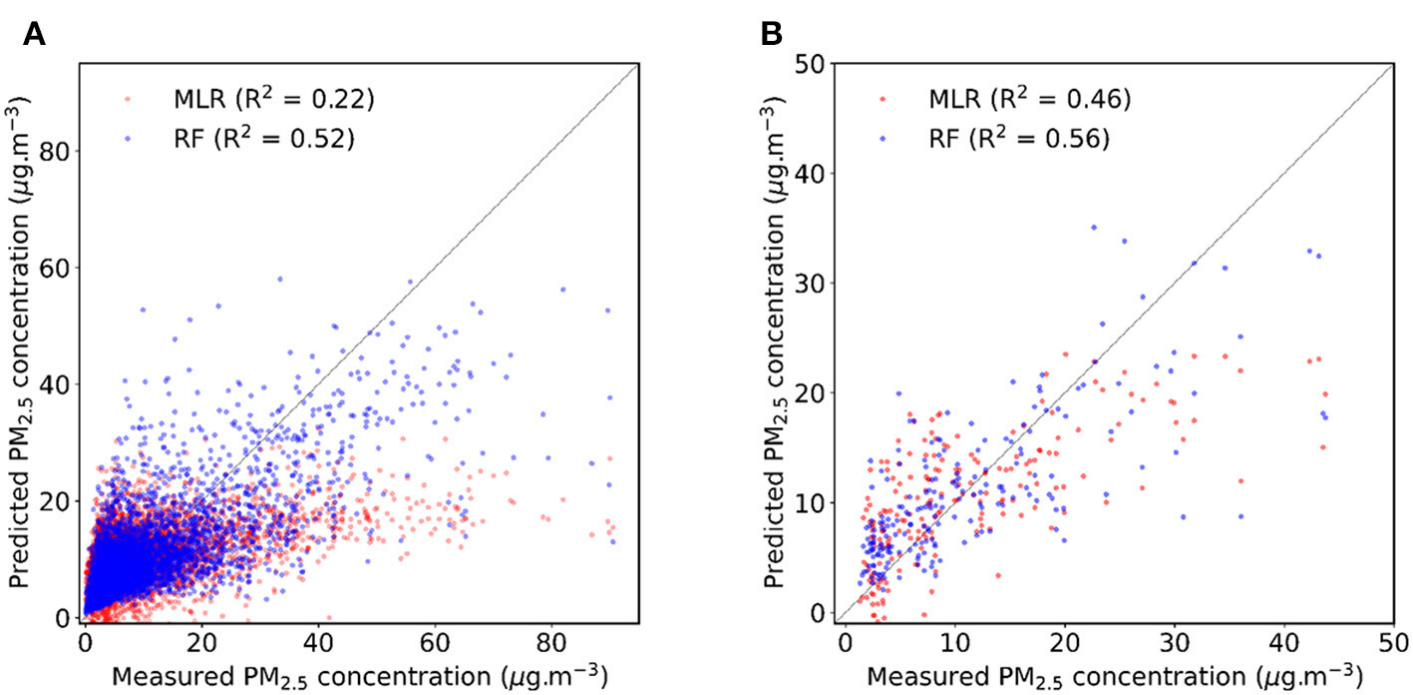
RF predictions against measured PM_2.5_ concentration in Curitiba for the validation period considering: **(A)** hourly averages and **(B)** daily averages.

**Figure 4:**
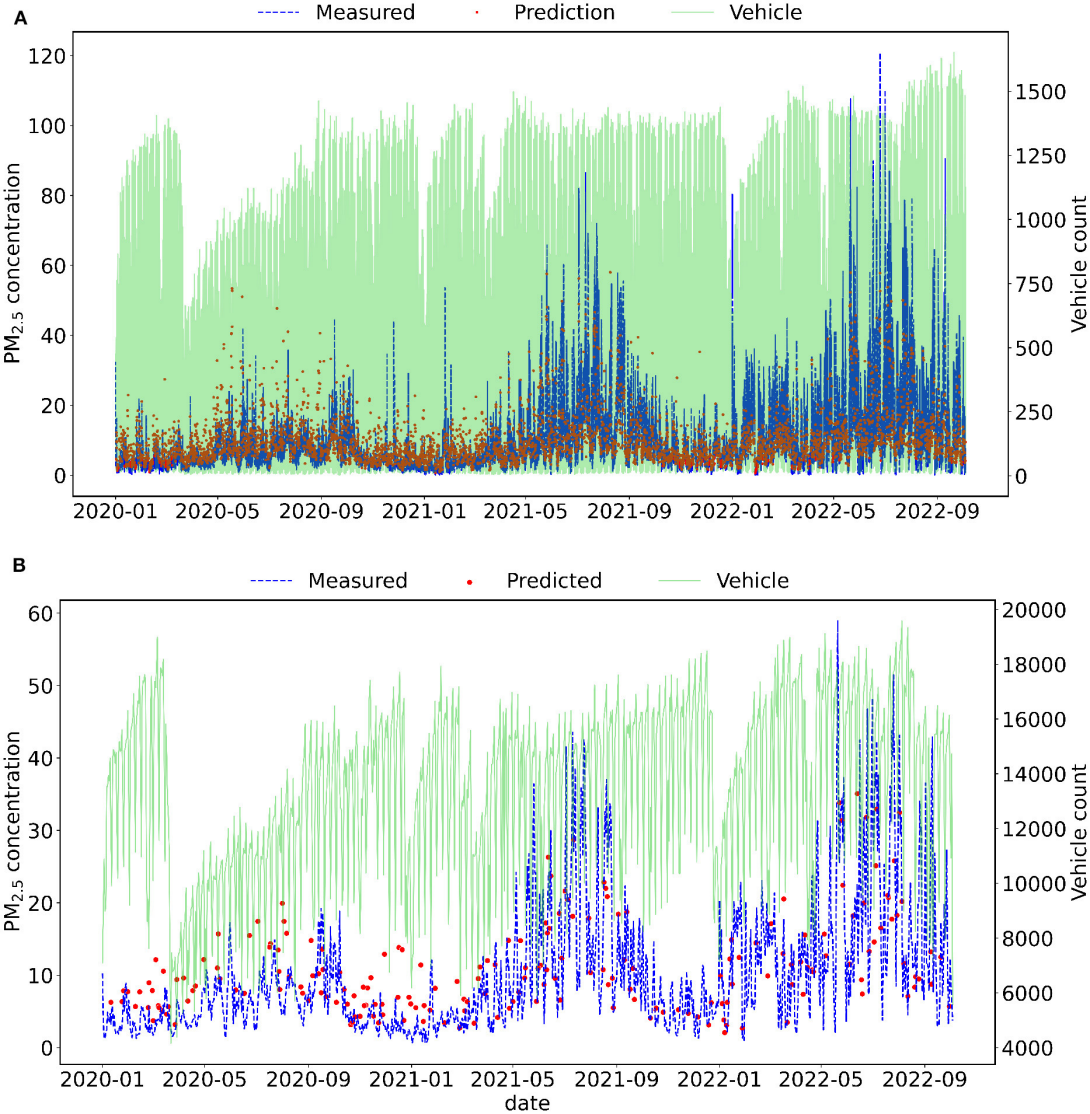
Test data time series of measured and predicted PM_2.5_ concentration in Curitiba using RF. Fine particles and vehicle count data were resampled using **(A)** hourly and **(B)** daily averages.

Figures 3, 4 show the RF results of test data for hourly and daily averages. Figure 3 presents a dispersion plot with predicted values on *x*-axis and measured values on *y*-axis, with the 1:1 line, and Figure 4 also shows the time series of predicted and measured PM_2.5_ concentration and the vehicle count data on the right y-axis. Figure 4 shows a decrease in the number of vehicles in the city of Curitiba in 2020, which probably contributed to the observed decrease in PM concentration.

RF model accuracy was higher compared with MLR in all cases, as well as MAE, MAPE, and RSME errors decrease and R^2^ value increase. The daily average of the RF model provided the highest accuracy value, reaching 99.37%. For both time scale predictions, there was a reduction in errors and an increase in the R^2^ value and accuracy using the RF model. By using the RF model, there was a greater increase in accuracy compared with MLR on the hourly scale, increasing the value by 0.46%, while the increase on the daily scale was only 0.07%. The increase in the R^2^ value and the decrease in errors when using the RF model were also more noticeable on the hourly scale than on the daily scale.

**Figure 5:**
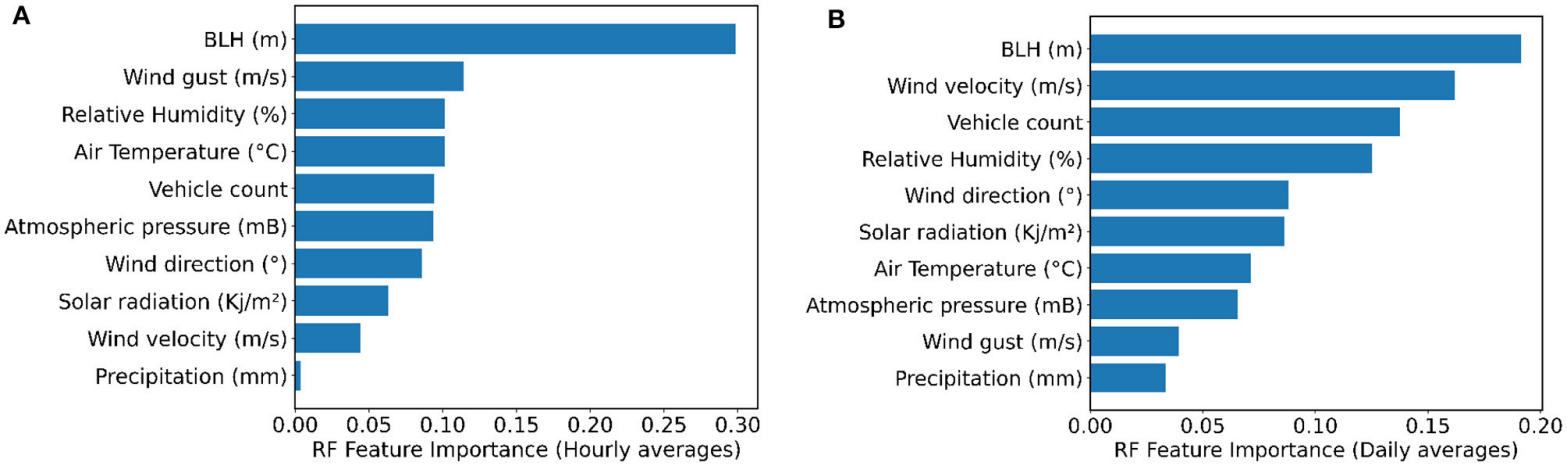
RF importance of the input variables for predicting PM_2.5_ concentration in Curitiba considering: **(A)** hourly averages and **(B)** daily averages.

The RF algorithm was able to select the most important variables for predicting PM_2.5_. Figure 5 shows the most important predictors used by RF model for hourly and daily averages. The importances in RF are computed as the mean and standard deviation of accumulation of the impurity decrease within each tree.

Boundary layer height was the most important variable, and relative humidity was the third most important for both time scale predictions: hour and day. There was a distinction between the other variables considering the hourly and daily averages, especially wind gust and wind speed, which exchanged between the second and second-to-last most important variables. Precipitation remained the least important variable, and vehicle count rose from the fifth most important variable to the third in the analysis of daily averages.

Initially analyzing the hourly average, the accuracy value for the LSTM model was very close to Naive's, but it was found that the accuracy, R^2^, and associated errors changed according to the size of window (look back period) and the number of hidden layers. It was also checked whether increasing the number of input variables could improve the performance of LSTM compared with Naive for hourly and daily scenarios, which are described in the following.

**Figure 6:**
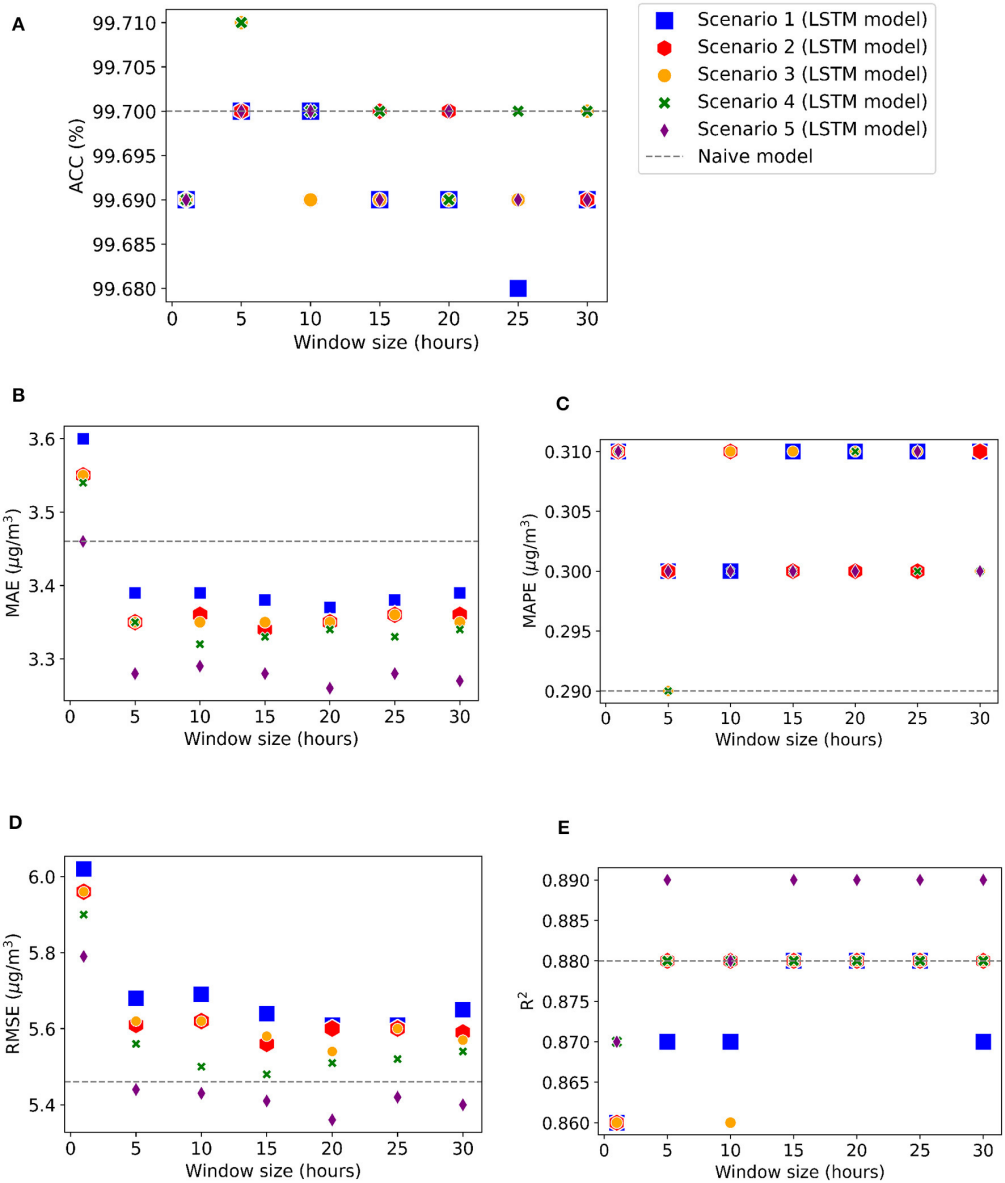
LSTM 1-h forecast performance metrics for different input scenarios as we increased the looking back window size. The metrics are: **(A)** Accuracy **(B)** MAE **(C)** MAPE, **(D)** RMSE and **(E)** R^2^.

In hourly timescale scenarios, the most important variables found in the RF model were added in stages as inputs to different LSTM models, starting with the BLH. Variables of equal importance were added in a single step, for example, relative humidity and temperature. Table 2 explains the different scenarios (H1, H2, H3, H4, and H5), and input variables were added to the LSTM models. Figure 6 shows the evolution of errors as the look back period increases for the five different inputs scenarios using three hidden layers, hidden dimension of 64, and 150 epochs of training.

**Table 2:** Description of scenarios and input variables used in each LSTM hour forecast model.

**Scenario**	**Input variables (hourly averages)**
H1	PM_2.5_
H2	PM_2.5_, BLH
H3	PM_2.5_, BLH, wind gust
H4	PM_2.5_, BLH, wind gust, relative humidity, air temperature
H5	PM_2.5_, BLH, wind gust, relative humidity, air temperature, vehicle count, precipitation, wind direction

The highest accuracy (99.71%) was found in scenarios H3 and H4 with a window size of 5, as observed in Figure 6A. Figure 6C shows that the lowest MAPE, 0.29%, value was found for the Naive model and LSTM with scenarios H4 and H5, while the other values varied between 0.30 and 0.31%. R^2^ reached a maximum value of 0.89 on all occasions for scenario H5 with a window size greater than or equal to 10, as shown in Figure 6E. Figure 6B shows that the MAE errors remained below the MAE value found in the Naive model for an LSTM window size equal to or greater than 5, and Figure 6D shows that the RMSE values were always lower by using LSTM, compared with Naive, for scenario 5 with a window size equal to or greater than 10.

Similar to the analysis of hourly averages, the most important variables found in the RF model with daily averages were added to the LSTM model, maintaining the same window size and using 3 hidden layers, hidden dimension of 64, and 150 epochs for training. Table 3 describes each scenario for the daily time scale (D1, D2, D3, D4, and D5), the input variables, and the LSTM and Naive model's evaluation metrics.

**Table 3:** Summary of Naive and LSTM model's performance results for different scenarios using daily averages of input variables and window size equal to 1 day.

**Scenario**	**Input variables**	**MAE(μg/m^3^)**	**MAPE (%)**	**RMSE (μg/m^3^)**	**R^2^ (-)**	**ACC (%)**
**Naive model**						
-	PM_2.5_	7.22	0.56	9.65	0.41	99.44
**LSTM model**						
D1	PM_2.5_	6.82	0.52	9.03	0.39	99.48
D2	PM_2.5_, BLH	6.80	0.52	8.85	0.41	99.48
D3	PM_2.5_, BLH, wind velocity	6.75	0.50	8.94	0.42	99.50
D4	PM_2.5_, BLH, wind velocity, vehicle count,	6.91	0.52	9.18	0.38	99.48
D5	PM_2.5_, BLH, wind velocity, vehicle count, relative humidity	6.83	0.53	8.89	0.41	99.47

**Figure 7:**
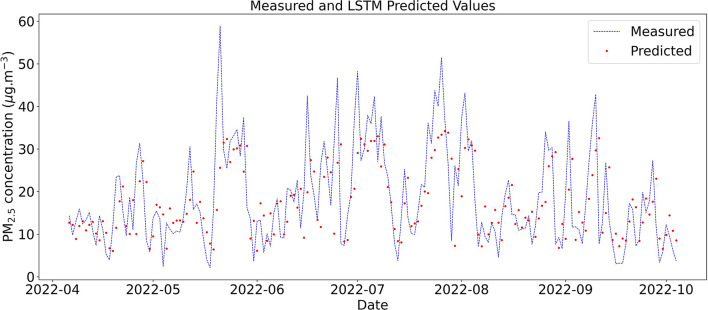
Test data time series of measured and predicted daily concentration of PM_2.5_ in Curitiba using LSTM with window size equal to 1 day.

For daily scenarios, the accuracy and R^2^ values were higher for the LSTM model, and MAE, MAPE, and RSME errors were lower when compared with the Naive approximation. When the number of inputs was increased with a window size of 1 day, there was a variation in the error and accuracy values. The highest accuracy and lowest MAPE were obtained in scenario D3 using three inputs: PM_2.5_ concentration, boundary layer height, and wind velocity. Figure 7 shows measured PM_2.5_ concentration and predicted concentrations using LSTM on a daily scale with PM_2.5_ concentration as input (scenario D1).

In 2022, vehicle count and particulate matter concentrations were higher than in 2021 and 2020. This behavior is associated with the COVID-19 pandemic, in which vehicle circulation decreased due to the lockdown in the City of Curitiba. Consequently, there was a decrease in PM_2.5_ concentration peaks during 2020–2021. Even though the LSTM was trained with lower values, corresponding to the period of the pandemic, the model was able to forecast the PM_2.5_ concentration in 2022.

## 4 Conclusion

The data gathered in this research provide important information about the City of Curitiba, Brazil, especially the relationship between number of vehicles and concentration of fine particles. Using the dataset created, the models for prediction and forecasting indicated that Random Forest and LSTM model were good estimators of PM_2.5_ concentration.

Model's performance was analyzed using measured data from Curitiba, while several inputs were tested. RF had better results for prediction compared with MLR, reaching 99.37% of accuracy at daily time scale. The lowest accuracy in prediction was the one that considered the hourly time scale using MLR. In general, models at daily scale performed better compared with models at hourly scale. The RF model identified boundary layer height as the most important input variable for both time scales and precipitation as the less important. Variations in wind conditions, vehicle count, air temperature, and relative humidity contributed significantly to predictions at hourly and daily scales.

The inputs recognized as most important in RF prediction (BLH, wind intensity, humidity, and vehicle count) were also important for LSTM forecast. The results of the LSTM model showed sensible variation depending mainly on model's looking back window size and inputs, sometimes reaching or exceeding the values found in the Naive model, with a maximum accuracy of 99.71% found on hourly scale with window size equal to 5 h. LSTM model had better performance compared with Naive's in forecasting at daily scale. Because of its ability to exploit the sequential nature of the data, LSTM network have the tendency to outperform Naive model.

Data showed the influence of COVID-19 pandemic on vehicle circulation and fine particulate matter concentration in Curitiba, with lower values in 2020 and 2021, followed by an increase in 2022. LSTM neural network was trained with pandemic data and was able to generate good forecasts for PM_2.5_ concentration in 2022, a post pandemic period.

RF and LSTM proved to be good models for the prediction of fine particles and forecasting in Curitiba, respectively. Our results help the physical understanding of factors influencing pollutant dispersion from vehicle emissions at the lower atmosphere in urban environment. As a suggestion for future studies, we recommend the application and comparison of other models to predict and forecast PM_2.5_, as well as testing larger window sizes to verify if it is possible to improve the performance of the model. It is also suggested to include vehicle information categorized by type or fuel as input variables of the models.

## Data availability statement

The original contributions presented in the study are included in the article/supplementary material, further inquiries can be directed to the corresponding author.

## Author contributions

MC: Conceptualization, Data curation, Formal analysis, Investigation, Methodology, Software, Validation, Visualization, Writing – original draft, Writing – review & editing. AS: Data curation, Resources, Writing – review & editing. EM: Conceptualization, Data curation, Formal analysis, Funding acquisition, Investigation, Methodology, Project administration, Resources, Software, Supervision, Validation, Visualization, Writing – review & editing. SN: Conceptualization, Formal analysis, Funding acquisition, Methodology, Project administration, Resources, Supervision, Validation, Visualization, Writing – review & editing.
